# Performance evaluation of a position-sensitive SiPM-based gamma camera for intraoperative imaging

**DOI:** 10.1186/s40658-026-00931-y

**Published:** 2026-08-17

**Authors:** Aramis Raiola, Fabio Acerbi, Cyril Alispach, Domenico della Volpe, Hossein Arabi, Alberto Gola, Habib Zaidi

**Affiliations:** 1 Départment de Physique Nucléaire et Corpusculaire (DPNC), Université de Genève, quai Ernest-Ansermet 24, 1211 Geneva, Switzerland; 2 Center for Sensor and Devices (SD), Fondazione Bruno Kessler (FBK), Via Sommarive 18, 38123 Trento, Italy; 3 Division of Nuclear Medicine and Molecular Imaging, Geneva University Hospital (HUG), 1211 Geneva, Switzerland; 4 Department of Nuclear Medicine and Molecular Imaging, University of Groningen, University Medical Center Groningen, 9700 RB Groningen, The Netherlands; 5 Department of Nuclear Medicine, University of Southern Denmark, DK-500 Odense, Denmark; 6 University Research and Innovation Center, Óbuda University, Budapest, Hungary

**Keywords:** Intraoperative gamma camera, Radio-guided surgery, Sentinel lymph nodes biopsy, Silicon photomultipliers

## Abstract

**Background:**

The POSiCS camera is a handheld and wireless, small field-of-view gamma camera developed for use in radio-guided surgery (RGS), with sentinel lymph node biopsy (SLNB) as its benchmark application. This compact and lightweight detector (about $350\,\textrm{g}$) maps tissues labeled with a ${^{99m}\hbox {Tc}}$ radiotracer to highlight target lesions. By enabling intraoperative visualization close to the surgical field, POSiCS aims to reduce invasiveness and operative time, improve localization accuracy, and minimize post-operative complications.

**Methods:**

The performances of two hardware configurations were tested: high-sensitivity and high-resolution, to meet application-specific needs. Tests assessed key parameters, including extrinsic spatial resolution, energy resolution, and sensitivity, as well as shielding effectiveness and response to different gamma energies. Characterization of the camera’s sensitivity and energy resolution employed a Flood (flat) ${^{99m}\hbox {Tc}}$ source, while the determination of spatial resolution and lateral collimator penetration was performed using a ${^{57}\hbox {Co}}$ point source. The response to ${^{177}\hbox {Lu}}$ was also investigated, given its growing role in theranostics, by imaging a cavity filled with the radionuclide and evaluating the reconstruction performance of POSiCS across its different gamma-ray emissions..

**Results:**

In direct contact, the spatial resolution was $1.9\pm 0.1\,\textrm{mm}$ in high-sensitivity mode and $1.4\pm 0.1\,\textrm{mm}$ in high-resolution mode. System sensitivity at $2\,\textrm{cm}$ source-detector distance was $481\pm 14\,\mathrm {cps/MBq}$ and $134\pm 8\,\mathrm {cps/MBq}$, respectively. Both modes showed an energy resolution of about 20% at 140 keV, though the high-resolution collimator exhibited increased scatter due to its larger tungsten content.

**Conclusion:**

The POSiCS camera combines compactness, ease of use, and reliable performance, making it suitable for RGS. Its interchangeable configurations allow balancing between sensitivity and resolution depending on surgical needs. The results demonstrate millimeter-scale resolution and adequate sensitivity for ${^{99m}\hbox {Tc}}$, confirming its suitability for intraoperative use. Imaging with ${^{177}\hbox {Lu}}$ was also proven possible, although achieved only with the 55 keV and 113 keV photopeaks. These outcomes support the camera’s potential for broader clinical deployment and further development toward advanced intraoperative nuclear imaging.

## Introduction

Radio-guided surgery (RGS), including intraoperative techniques, such as sentinel lymph node biopsy (SLNB) and radioguided occult lesion localization (ROLL), is a widely adopted approach, particularly in the surgical management of breast cancer and cutaneous melanoma. [1, 2]. Despite the availability of well-established alternatives to RGS (such as Fluorescence-Guided Surgery (FGS) or Ultrasound-Guided Surgery), the preoperative administration of radio-labeled molecules remains one of the most widely used techniques for intraoperative localization of oncological lesions. This is due to the large mean free path of gamma rays in living tissues, which allows for the direct detection and localization of activity hot-spots from outside the patient’s body [1–3].

The state-of-the-art practice in SLNB and ROLL is based on radiation-counting probes to localize lesions or sentinel lymph nodes (SLNs) [4, 5]. Although this approach has been proven reliable by clinically comparing preoperative lymphoscintigraphies with the number of SLNs localized through a probe-based search, probes still inherently bear a number of drawbacks. To address this shortcoming, an increasing number of 2D Intraoperative Gamma Cameras (IGCs), also known as Small Field-of-View (SFOV) gamma cameras, have been developed in the last two decades [6, 7]. This trend has been thoroughly studied in recent years, thanks to the comprehensive reviews in the field of IGCs, such as [4] and a more recent follow-up [5]. The main advantages of these cameras are their imaging capabilities, which allow surgeons to localize hot spots near the “blast zone" (radiotracer injection site) and to provide a method to verify post-excision images to ensure that the target lymph node or lesion was entirely resected [8, 9]. Additionally, imaging is intuitive and does not require the specific and lengthy training needed to become proficient with probes. It has also been shown that 2D imaging of oncological lesions can reduce surgery duration, as it facilitates the localization of target tissues more quickly [8, 10].

POSiCS (Position-sensitive SiPM Compact and Scalable gamma-Camera) is a handheld, compact, and wireless gamma camera designed for real-time intraoperative guidance. The device is based on a pixelated LYSO:Ce scintillator and a 3*× *3 array of position-sensitive SiPMs (LG-SiPMs). This module is equipped with two interchangeable collimators: a short septa, low-energy, high-sensitivity (LEHS) collimator and a longer, low-energy, high-resolution (LEHR) collimator, enabling dual operating modes that can be selected intraoperatively to adapt to different clinical needs. Designed to effectively detect photons at $140.5\,\textrm{keV}$, corresponding to the emission energy of $^{99\textrm{m}}\textrm{Tc}$ [11], the standard radionuclide used in RGS, POSiCS exhibits enhanced performance relative to existing systems.

In this work, we present the results of the first performance studies on the prototype POSiCS camera module. In the "Materials and methods" section, we present the experimental methods adopted to measure the main performance parameters of the camera with both collimators. The tests were inspired from the 2023 NEMA standards for gamma camera qualification [12]. However, as these standards are primarily designed for large clinical scanners, only a subset of the proposed measurements and procedures applies to our device, given its specific design and use case. The results of this series of procedures are displayed and commented in the following section. Conclusively, in the "Discussion" section, we put into prospective the obtained results with current literature reviews about IGCs.

## Materials and methods

### The POSiCS camera

**Fig. 1:**
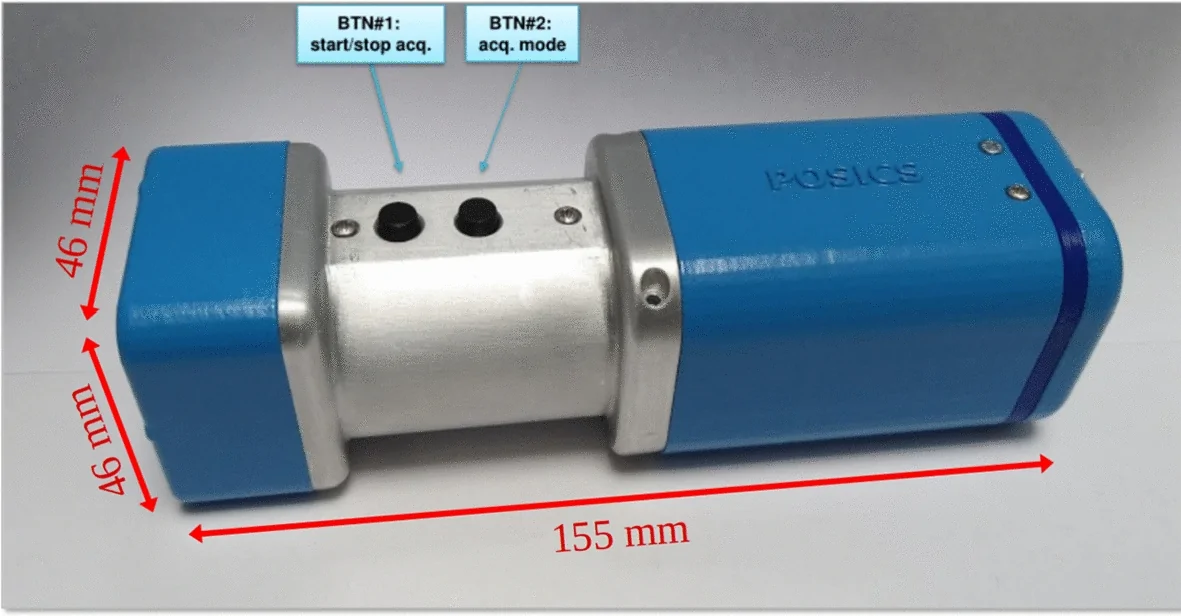
Prototype module of the POSiCS gamma camera equipped with the LEHR collimator

The current POSiCS camera module has a total length of 147 mm (155 mm with the LEHR collimator mounted), with a Field-of-View (FOV) of $30.8\times 30.8~\mathrm {mm^2}$. From this, we define a Useful FOV (UFOV) of $28.6\times 28.6~\mathrm {mm^2}$. The module total weight is 313 g (with the LEHS collimator) and 381 g (with the LEHR collimator). Further technical details about the camera are provided elsewhere [13]. A picture of the camera’s prototype wireless module is shown in Fig. 1.

#### Collimator geometry

The POSiCS camera is equipped with two interchangeable parallel-hole collimators: one targeting high spatial resolution (LEHR) for surgical procedures that require precise identification of target lesion margins, and the other designed to achieve high sensitivity (LEHS) for faster scanning, even at low injected activities. The different parameters that qualify both objects were chosen through theoretical calculations and simulations using GATE [14].

Due to its high atomic number (implying a short attenuation length) and good resistance to shocks [5], tungsten was chosen. All collimator parameters were chosen to ensure a septal penetration probability of less than *5%* [15]. The collimator was designed with external shielding walls that extend beyond the collimator holes and support the scintillator, reducing the amount of gamma rays entering from the side of the camera.

#### LYSO scintillator approach

The radiation-sensitive component of the POSiCS camera is a pixelated LYSO:Ce scintillator. This Lutetium-based crystal is typically used in Positron Emission Tomography (PET) scanners due to its fast decay time ($\sim 40~\textrm{ns}$), high light yield ($\ge 32000~\mathrm {ph~MeV^{-1}}$), and high density [16]. The camera was therefore built using a very thin crystal (a few millimeters thick), keeping a high photoelectric absorption probability at $140.5~\textrm{keV}$. The scintillator is pixelated in a 30*× *30 matrix. Each pixel is coated with a BaSO$_4$ reflector to increase light collection on the light detector.

**Fig. 2:**
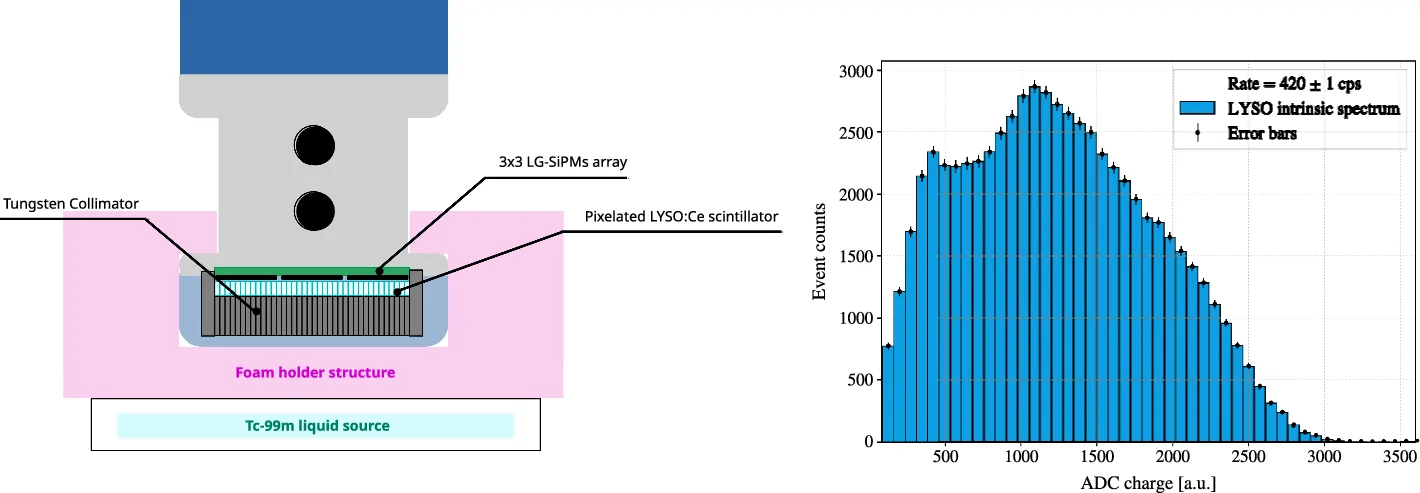
*Left *Scheme of the flood map setup used in this work. The estimated distance from the collimator entrance to the center of the cavity is 2 cm. *Right *LYSO:Ce scintillator intrinsic radioactivity spectrum registered with POSiCS. The structure of the spectrum depends on the crystal volume and shape, which in turn determines the probability of absorbing the emitted *γ *-rays (in coincidence). The long tails are due to the absorption of the initial *β * particle and are therefore shaped after the *β *-emission spectrum [18]. $\mathrm {^{99\,m}Tc}$ photoepeak is expected to lie at ADC channel 460

A peculiarity of LYSO:Ce is its intrinsic radioactivity due to the natural 2.6% abundance of $^{176}$Lu isotope, which decays into $^{176}$Hf through a $\beta ^{-}$ decay. The process is followed by the emission of three gamma rays at energies: $88~\textrm{keV}$, $202~\textrm{keV}$, and $307~\textrm{keV}$, which can interact within the crystal itself [17, 18]. The presence of this isotope establishes a background activity within the camera FOV at about $420~\textrm{cps}$, which is mainly eliminated through energy window selection and subtracted during image correction. This feature is often cited as being suited for self-calibration in PET scanners and can represent an advantage in this direction in the future [19]. The LYSO:Ce intrinsic spectrum reconstructed with the POSiCS camera is displayed in Fig. 2.

#### LG-SiPMs photodetector

Since 2013, reviews in the field of IGCs showed a trend toward implementing Position-Sensitive (PS-) PMTs in SFOV gamma cameras [4]. At the same time, most recent reviews highlight a shift in the past decade toward using SiPMs for the optical readout of scintillator crystals in IGCs [5]. This transition is driven by the advantages of solid-state detectors, including low power consumption, compactness, high quantum efficiency, and insensitivity to magnetic fields.

In the current context of light detectors for IGCs, POSiCS proposes an innovative approach by moving towards PS-SiPMs, leveraging the new Linearly Graded SiPMs technology (LG-SiPMs) designed and produced by FBK [20–22]. Through an array of 3x3 LG-SiPMs with peak sensitivity at $420~\textrm{nm}$, an active area of about $10\times 10~\mathrm {mm^2}$ per sensor, and microcell pitch of $25~\mathrm {\mu m}$, it is possible to reconstruct the position of each scintillation event, through a weighted charge (and amplitude) distribution towards the output channels. Each Single Photon Avalanche Diode (SPAD) composing the SiPM is connected to a curent divider (one for the X and the other for the Y coordinates) and a resistive network, which performs a light spot CoG reconstruction, encoded in the relative amount of charge output by each readout channel [13, 20]. This Anger CoG reconstruction is typically used between SiPMs or PMTs in standard gamma cameras, but this approach is implemented at the single microcell level within the POSiCS camera. The 3*× *3 array configuration, with the “smart channel" approach (see [20]), allows CoG reconstruction with only 8 readout channels: 4 encoding for the x coordinate and 4 reconstructing the y coordinate. More specifically, $x_1-x_4$ and $y_1-y_4$ refer to the signals current measured at the four x- and y-encoding outputs of the LG-SiPMs array, respectively. These signals are combined to reconstruct the interaction position, producing normalized coordinates (*x*, *y*) in the range [− 1,1], according to:

1a $$\begin{aligned} x_{pos}&= \frac{x_1+\frac{1}{3}(x_3-x_2)-x_4}{x_1+x_2+x_3+x_4}, \end{aligned}$$

1b $$\begin{aligned} y_{pos}&= \frac{y_1+\frac{1}{3}(y_3-y_2)-y_4}{y_1+y_2+y_3+y_4} \end{aligned}$$

The weighted position calculation is performed directly within the camera digital electronics, which in turn sends the reconstructed positions through a wireless connection. This photon detector demonstrated exceptional intrinsic spatial resolution, achieving the reconstruction of structures separated by a few hundred microns [20, 23–25].

The limited number of channels enables a fast and straightforward readout, allowing for wireless data transmission to the computer before applying the necessary image quality processing.

**Fig. 3:**
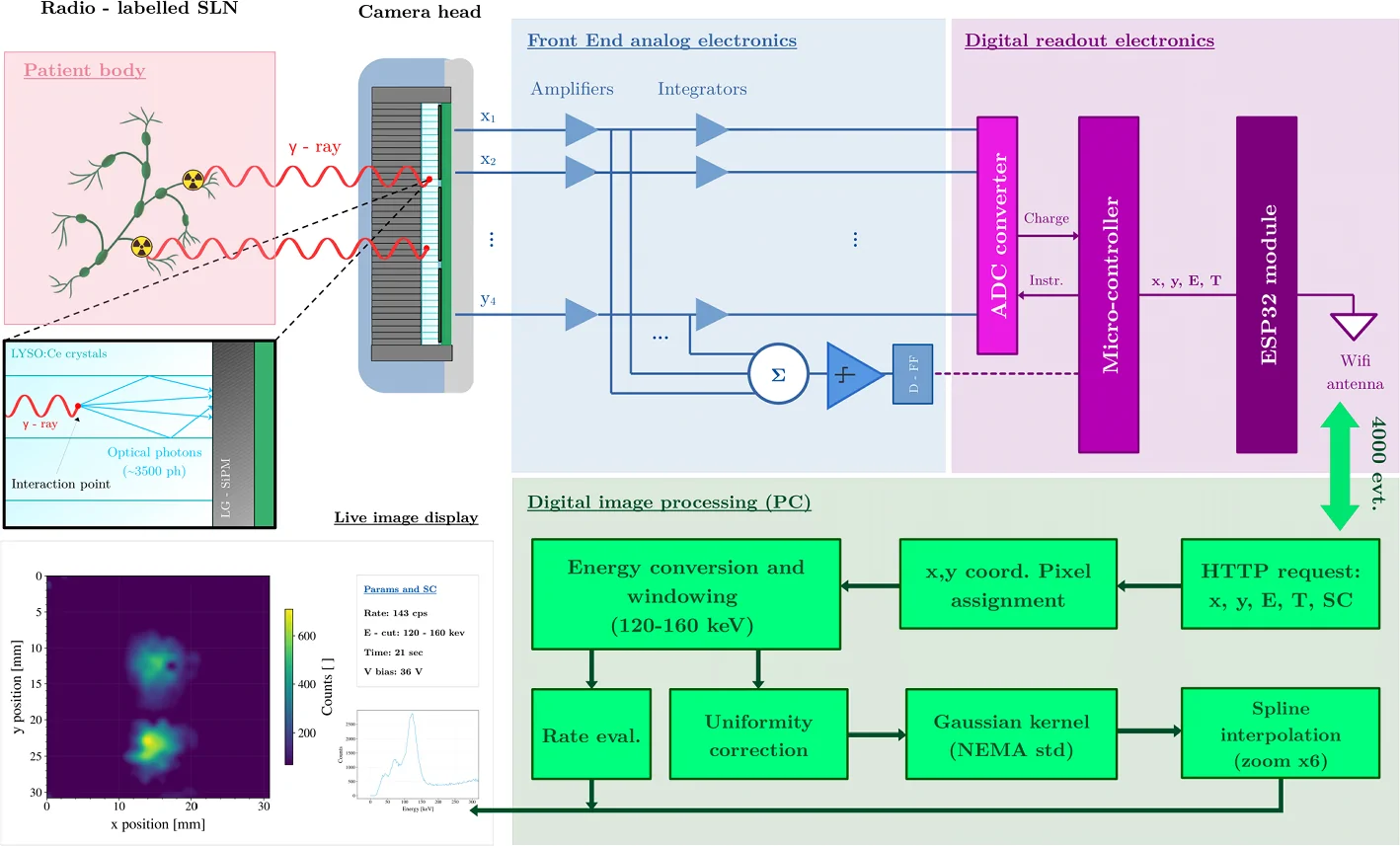
Scheme of the full pipeline of the POSiCS camera, from the gamma-ray emission by radio-labelled tissues, to the final image treatment and enhancement, with the possibility of live displaying the reconstructed image

#### Approach to event reconstruction

After a scintillation event in the scintillator, the eight SiPM signals are amplified by transimpedance amplifiers (TIAs). Their summed charge output is sent to a comparator, which triggers the acquisition of the 8 signals only when the user-selectable threshold is crossed, in an event-driven acquisition approach. The resulting per-channel charge is integrated and digitized by an eight-channel ADC. The microcontroller then computes the event energy *E* (sum of all channels output charge), timestamp *T*, and the *x*- and *y*-coordinates using Eq. 1, and also provides slow-control (SC) data such as bias voltage, current, temperature, and battery status (see Fig. 3).

Each event is stored as *(x, y, E, T)* and transmitted to an external computer via an ESP32 module in packets of 4000 events or after a user-defined time window has elapsed. The data is retrieved via HTTP GET requests. The camera settings are configured through the same ESP32 using HTTP POST commands to the DACs. For further technical details about the POSiCS camera electronics, see [13].

Reconstructed images are corrected using a three-step calibration and correction procedure. First, once the incident position of a gamma ray is determined (x,y CoG coordinates), the event is assigned to the corresponding camera pixel (scintillator crystal, 1.1 mm pitch) associated with the scintillator crystal encompassing the interaction position. This spatial mapping is established through a flood-field calibration scan, which serves as the primary calibration step before operating the camera.

Second, a pixel-by-pixel energy calibration is performed. In this step, a scaling coefficient is applied to convert the SiPMs charge response into a physical energy. Due to non-uniform light collection across certain regions of the FOV, as well as slight variations in the gain and response characteristics of the SiPMs forming the optical readout array, each pixel is assigned an individual conversion coefficient to convert the digitized charge to a physical energy in keV. Following this step, a user-defined energy window can be applied in software to selectively retain events consistent with photoelectric interactions, thereby suppressing contributions from Compton-scattered events that occur either within the LYSO crystal or in the surrounding environment.

Finally, a uniformity correction is applied to the reconstructed image to compensate for spatial sensitivity variations. This step enhances the signal in regions typically exhibiting under-response, such as those corresponding to interstitial gaps between SiPMs or at the periphery of the camera field of view, thereby ensuring a more homogeneous image representation.

With this acquisition system, it is possible to display images live with a delay of the order of 1 s. The final images can also be processed using a 2D-Gaussian kernel convolution to enhance the reconstruction uniformity, following the guidelines presented in the 2023 NEMA standards for the validation of gamma cameras [12]. Finally, the smoothed image is interpolated using splines of order 2.

### Performance evaluation

The POSiCS camera underwent extensive performance testing to evaluate the reliability of its image reconstruction algorithms and to characterize its applicability across different imaging scenarios. The key performance parameters investigated included spatial resolution, sensitivity, and energy resolution. These evaluations required the use of both point-like and extended radioactive sources.

To assess spatial resolution, a point source, defined as having a diameter smaller than that of the scintillator pixel, was necessary. Due to the practical limitations of producing such a source with $\mathrm {^{99\,m}Tc}$, a solid $\mathrm {^{57}Co}$ source was employed. This isotope emits gamma-rays at 122 keV, serving as a suitable surrogate for the 140.5 keV photons of $\mathrm {^{99\,m}Tc}$.

Conversely, the extrinsic sensitivity and energy resolution tests with $\mathrm {^{99\,m}Tc}$ were performed using a custom-designed flat liquid phantom that provided uniform irradiation of the detector, as illustrated in Fig. 2. Finally, additional tests were conducted with $\mathrm {^{177}Lu}$, a radionuclide used in theragnostics that emits multiple photon energies ranging from low-energy X-rays to *γ * emissions above 300 keV. These measurements aimed to evaluate the camera’s reconstruction performance with multi-energy emissions and to determine its suitability for imaging with $\mathrm {^{177}Lu}$. A summary of the used radiotracers for the performance evaluation is presented in Table 1

**Table 1:** Summary of the radioactive sources and test conditions used to evaluate the POSiCS camera performance

Isotope	Energy [keV]	Window [keV]	Source form	Test
$\mathrm {^{57}Co}$	122	100–140	Sealed point source	Spatial resolution, collimator leakage
$\mathrm {^{99\,m}Tc}$	140.5	120–160	Flat liquid phantom	Sensitivity, Energy resolution
$\mathrm {^{177}Lu}$	55–208	Multiple	Liquid source	Multi-energy reconstruction

#### Extrinsic spatial resolution

The extrinsic spatial resolution was assessed by placing a point-like $^{57}$Co source ($E_\gamma =$122 keV) in the center of the camera’s FOV. In this position, 100,000 events were acquired, and an image was reconstructed following the correction pipeline described in "The POSiCS camera" subsection. No smoothing or kernel convolution is applied to assess the device’s performance without digital image quality enhancement. An energy cut window between 100 and 140 keV was applied to reject scattered photons (cf. Fig. 5). The obtained reconstructed *x* and *y* profiles were fitted with a Gaussian profile, and their full width at half maximum (FWHM) was calculated. We define the device’s resolution as the average FWHM between the two directions.

The uncertainty on the resolution is the summation in quadrature of the statistical error (obtained through the covariance matrix of the fit) and a systematic uncertainty. Systematics is defined as the variation in resolution due to the different behavior of the camera in other areas of the FOV. The systematic uncertainty is evaluated through a scan of 15 positions on the device’s FOV with the $^{57}$Co point source, acquiring 100,000 events at each position. Each reconstructed spot is fitted with a Gaussian profile, from which the FWHM is extracted. The standard deviation of this sample of 15 measured resolution values represents the systematic error of the resolution measurement.

The measurement at the center of the FOV was repeated for different source-to-collimator distances (from 0 to 45 mm in 5 mm increments) under two conditions: with and without scattering material placed between the source and the camera head. The chosen scattering material is Polymethyl methacrylate (PMMA). This approach enables us to estimate the expected resolution in biological tissues, whose density is comparable to that of PMMA. The obtained resolution is also compared with the theoretical spatial resolution [26, 27]

#### Sensitivity

The camera’s sensitivity is defined as the registered trigger rate divided by the activity of the source within the camera’s FOV. This parameter is strongly dependent on the geometry of the collimator, the efficiency of the scintillator crystal (i.e., the probability of absorbing a gamma ray through photoelectric effect at a defined energy), and the dead-time of the readout. Following the guidelines presented in the NEMA standards for gamma camera qualifications [12], we performed sensitivity measurements, ensuring that rates did not exceed 20,000 counts per second (cps).

The tests were performed by placing the flood phantom filled with sodium pertechnetate (${\hbox {N}_{\hbox {a}}\hbox {T}_{\hbox {c}}\hbox {O}_{4}}$) obtained from the ${^{99}\hbox {Mo}/^{99m}\hbox {Tc}}$ generator at the Geneva University Hospital (HUG) and dissolved in saline solution, to irradiate the FOV uniformly. Then, the rate was registered through the camera’s wireless readout, in packets of 4000 events. The activity of the source visible in the FOV was evaluated through the following conversion:

2 $$\begin{aligned} A_\mathrm{{FOV}}(t)=A(t_0)\cdot \frac{S_\mathrm{{FOV}}}{S_\mathrm{{FP}}} \cdot e^{-\frac{t\ln {2}}{\tau _{1/2}}} \end{aligned}$$

where $t_0$ is the source’s production time (when activity $A(t_0)$ was measured by a *4π * detector available at Geneva University Hospital, with a precision down to tens of kBq), $\tau _{1/2}=6.02~\textrm{h}$ is the half-life of metastable $\mathrm {^{99\,m}Tc}$, $S_\mathrm{{FOV}}$ is the area of the FOV, and $S_\mathrm{{FP}}$ is the surface area of the Flood Phantom. The sensitivity over time is therefore computed as:

3 $$\begin{aligned} g(t)=\frac{R(t)}{A_\mathrm{{FOV}}(t)} \end{aligned}$$

Here, *R*(*t*) is the trigger rate of the camera at time *t*. The reported error on the sensitivity measurements is defined as the quadrature sum of the statistical uncertainty on the rate and the systematic uncertainty on the activity, giving the uncertainty in sensitivity. The latter is the propagated error of the initial injected activity in the phantom (set at *10%* of the initial activity) and the uncertainty on the custom-made phantom’s dimension:

4 $$\begin{aligned} \sigma _{sens}=\sqrt{\frac{R(t)}{\Delta t}}\oplus \sigma _{syst} \end{aligned}$$

where *Δ t* is the duration of the time bin during which the rate was evaluated. The procedure is repeated with the LEHS collimator and the LEHR collimator, placing the source at 2 cm from the camera’s lid. No background subtraction was performed, due to the high activity of the source with respect to the *∼ *500 cps rate from LYSO:Ce, which is reduced to few tens of cps after energy window selection.

#### Energy resolution

The energy resolution was evaluated for both the LEHR and LEHS collimators by placing the Flood phantom in front of the camera to uniformly irradiate the FOV at a distance of 2 cm from the source. The resulting spectrum was corrected using a pixel-by-pixel approach, following the device’s standard calibration procedure. The resolution was computed by fitting the photopeak (identified as the first and most prominent peak on the right, corresponding to the physical interaction process that leaves the most energy in the crystal) with a Gaussian profile and considering the resolution as its FWHM divided by the central theoretical energy of 140.5 keV.

#### Collimator side penetration

The camera’s external shielding was tested to assess the penetration of gamma rays through the tungsten collimator’s walls, both of which are designed to prevent external radiation from reaching the scintillator crystal. An IGC does not require strong directional sensitivity, unlike 1D gamma probes, which rely on it due to their lack of imaging capability. Directionality is, therefore, a key performance aspect typically studied in detail for probes [28]. In the case of POSiCS, a penetration study was performed to ensure that possible activity hotspots outside the FOV do not influence the imaging performance of the POSiCS camera. Moreover, penetration of gamma-ray from the side are ultimately visually identified and thus can be removed by truncating the image.

This test was performed by registering the detected rate of a $\mathrm {^{57}Co}$ point source in free air, positioned 3 cm from the center of the camera, at three distinct angles: one at $0^\circ$ relative to the normal of the FOV, and the other two at $45^\circ$ and $-45^\circ$ from the same normal direction. The 100–140 keV energy window (used throughout this work for $\mathrm {^{57}Co}$ data processing) was adopted.

The rate data was collected in 150 packets of 4000 events for each tested angle. For each packet, the initial and final timestamps are used to compute the average rate of that packet.

#### Energy response to other radiotracers ($\mathrm {^{177}Lu}$)

To test the camera’s performance at different *γ *-rays energies, we performed a study using $\mathrm {^{177}Lu}$ as a radiotracer. This isotope has been adopted in nuclear medicine due to its multiple applications in radiotherapy [29–33]. $\mathrm {^{177}Lu}$ is often linked to peptides (such as the case of $\mathrm {^{177}Lu}$-DOTA-TATE and $\mathrm {^{177}Lu}$-PSMA), to target cancerous cells in several radiotherapeutic procedures, aiming to treat prostate cancer, neuroendocrine tumors, bone metastasis, and other malignancies [31]. $\mathrm {^{177}Lu}$ nuclei decay into Hf-177 through $\beta ^{-}$ emission. Hf-177 nuclei live in a metastable state for characteristic times of a few hundred picoseconds (depending on the state), reaching stability through the emission of gamma rays [29]. The emission energies issued by the above-mentioned gamma dis-excitation: 208 keV (10.4% probability), 113 keV (6.2% probability), 55 keV (4.5% probability), and 65 keV (1.2% probability) [31, 33]. Some other, less frequent energies are also present.

To perform dosimetry measurements on patients during therapy, SPECT scanners have already been used to map the activity regions traced by $\mathrm {^{177}Lu}$ gamma-rays emission [30]. Moreover, SFOV gamma cameras were also proposed and tested for their response to $\mathrm {^{57}Co}$, due to the possibility of rapid scans with a smaller and more practical device [34]. The use of handheld cameras can also contribute to pharmacokinetic studies, allowing for faster and more frequent scans [35].

The performance of the POSiCS camera was evaluated by imaging a $204~\textrm{mm}^3$ cavity, representing the rear tumor volume within a BIOMETECH fillable mouse phantom [36], depicted in Fig. 9. The cavity was filled with 12 MBq of $\mathrm {^{177}Lu}$. The phantom was positioned in direct contact with the POSiCS camera, which was equipped with the LEHS collimator. Data were acquired over a total acquisition time of 100 s.

## Results

### Extrinsic spatial resolution

**Fig. 4:**
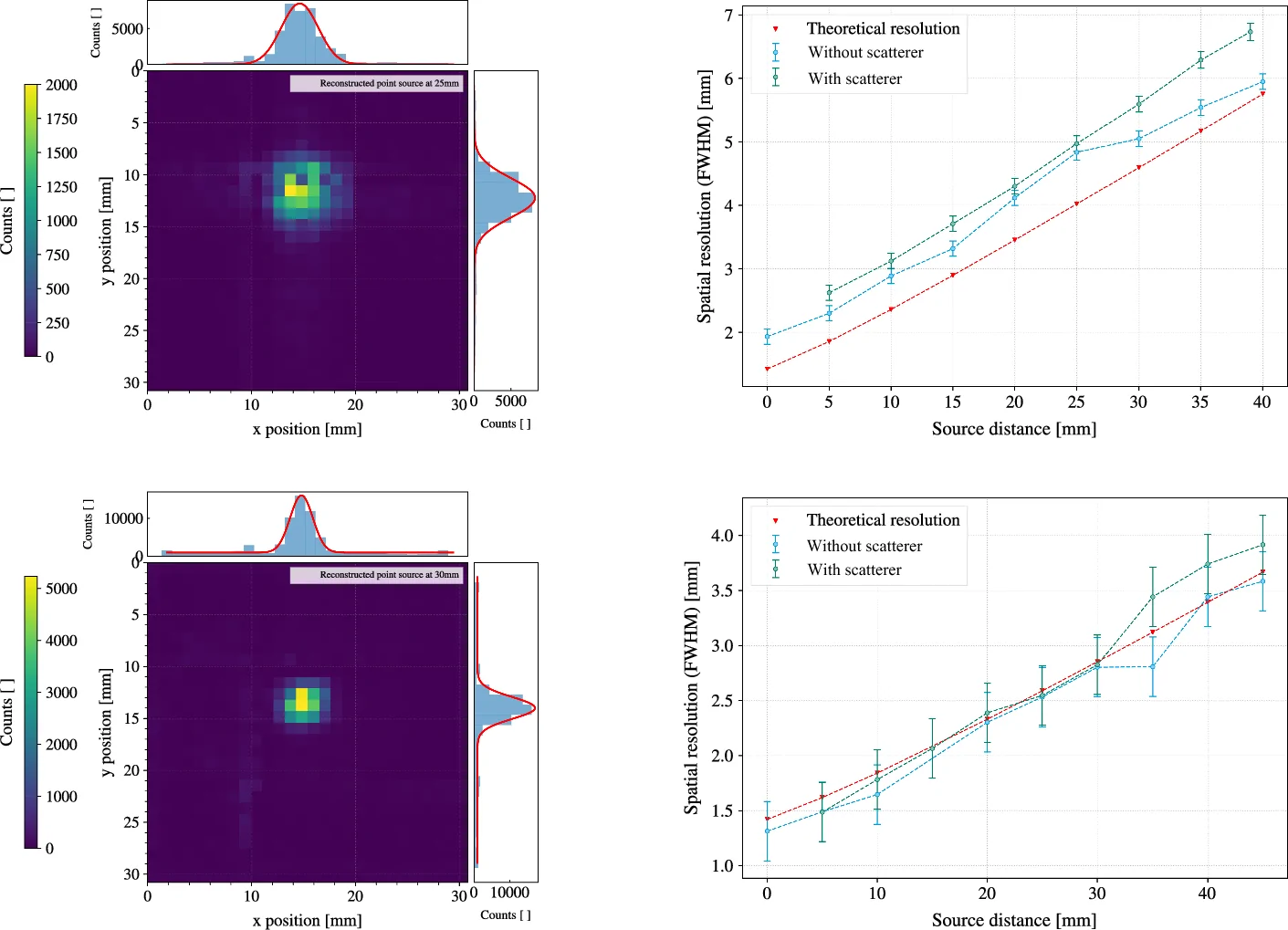
*Top left* Example of the reconstructed point source at 3 cm from the camera (free air) with LEHS collimator. *Top right* Resolution of the POSiCS camera with the LEHS collimator mounted on for different distances. *Bottom left* Example of the reconstructed point source at 3 cm from the camera (free air) with LEHR collimator. *Bottom right* Resolution of the POSiCS camera with the LEHR collimator

The results of the spatial resolution evaluation are presented in Fig. 4.

By placing the source in contact with the camera, we obtained a resolution with the LEHR collimator of 1.4 ± 0.1 mm, which increased to 2.8 ± 0.1 mm when the source was moved 3 cm away. In contact with the camera head, the LEHR and LEHS collimators show comparable resolutions. However, due to the well-known collimator projective effect [26], the resolution of the LEHS collimator degrades faster than for the LEHR collimator. With the LEHR collimator, we measured a spatial resolution of 1.9 ± 0.1 mm at 0 cm from the source, which increased to 5.1 ± 0.1 mm at 3 cm. Moreover, the difference between measurements in free air and with scatterer material is within error bars using the LEHR collimator, while the presence of PMMA worsens resolution with the LEHS collimator. This phenomenon is likely related to the collimator’s improved ability to reject photons scattered in the PMMA layers between the source and the camera.

**Fig. 5:**
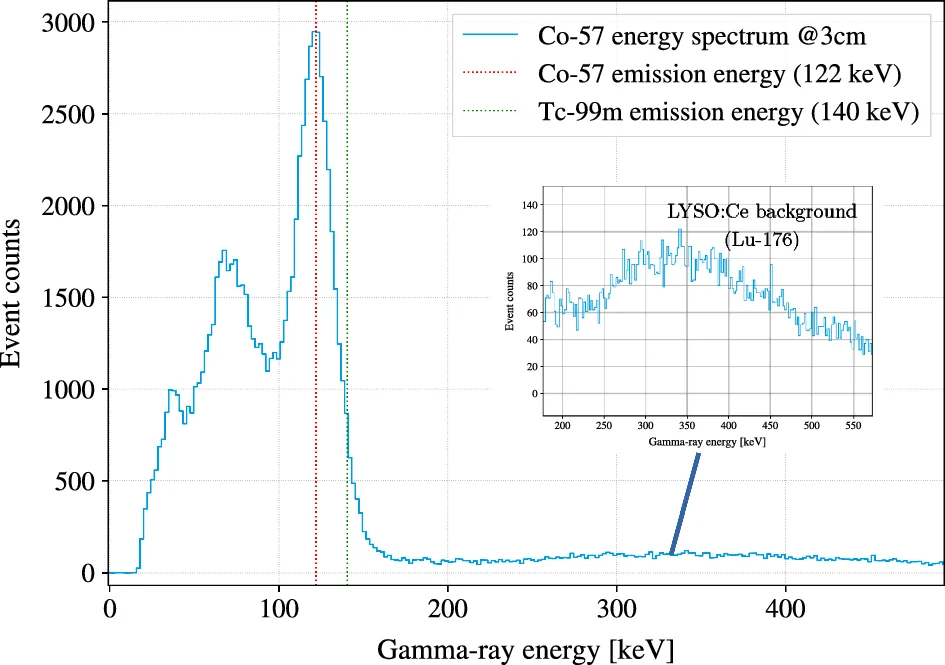
Example of energy spectrum of the Co-57 point source positioned 3 cm from the center of the camera FOV (LEHR collimator). The background-dominated region associated with LYSO intrinsic radioactivity is highlighted, illustrating that it does not significantly contribute to the energy window centered around 122 keV. For reference, the position of the $\mathrm {^{99\,m}Tc}$ emission line at 140.5 keV is also reported

For the LEHS collimator, the measured extrinsic resolution in free air is larger than the theoretical one by 15% on average, as displayed in Fig. 4 (top Right). This inconsistency, which lies beyond error bars, is mainly due to the light detector and the thin collimator septa. Theoretical calculations, indeed, do not consider the resolution spread due to the light detector, which, in our case, presents some deformations toward the interstitial gaps between SiPMs, for instance. Moreover, for a short collimator with thin septa, it is possible to have an increased septal penetration, and therefore a reduced spatial resolution, which is challenging to estimate empirically. Consequently, we can conclude that the camera’s extrinsic resolution aligns reasonably well with theoretical expectations, showing a similar slope as the distance increases. The LEHR collimator analysis reveals a more precise agreement with theoretical expectations, likely due to the reduced number of accepted scattered photons and reduced septal penetration. Due to the possibility of applying a Gaussian kernel convolution to smooth images, following the NEMA standards protocol, in Table 2 we provide a list of resolution for two source-collimator distances (0.5 cm and 3 cm), both with scattering material and in free air. We notice that the resolution is strongly worsened by the smoothing which makes the image appear more uniform, removing random or systematic intensity fluctuations in the FOV, but also increasing the size of the reconstructed point source.

**Table 2:** Measured spatial resolution after Gaussian kernel convolution for the LEHS and LEHR collimators under free-air and scatter conditions at source-to-collimator distances of 0.5 and 3 cm

	Free air		Scatter	
	0.5 cm	2 cm	0.5 cm	2 cm
LEHS Collimator	*3.8 ± 0.1*	*5.0 ± 0.1*	*4.0 ± 0.1*	*5.2 ± 0.1*
LEHR Collimator	*3.2 ± 0.3*	*3.7 ± 0.3*	*3.3 ± 0.3*	*3.9 ± 0.3*

### Extrinsic sensitivity

**Table 3:** Sensitivity results compared to the theoretical expectations

Sensitivity results	LEHS collimator	LEHR collimator
Sensitivity BEC [cps/MBq]	1444 ± 31	730 ± 16
Sensitivity AEC [cps/MBq]	481 ± 14	134 ± 8
Distribution kurtosis	2.89	2.97
Distribution skewness	0.04	0.00

BEC, before energy cut; AEC, after energy cut

For the camera equipped with the LEHS collimator, we report a sensitivity of $481\pm 14~\mathrm {cps/MBq}$ after rejecting all events whose energy does not lie within the spectral window adopted in this work for $\mathrm {^{99\,m}Tc}$ (120 keV-160 keV). Instead, we observe a significant reduction in sensitivity for the LEHR collimator, down to $134\pm 8~\mathrm {cps/MBq}$, as expected.

**Fig. 6:**
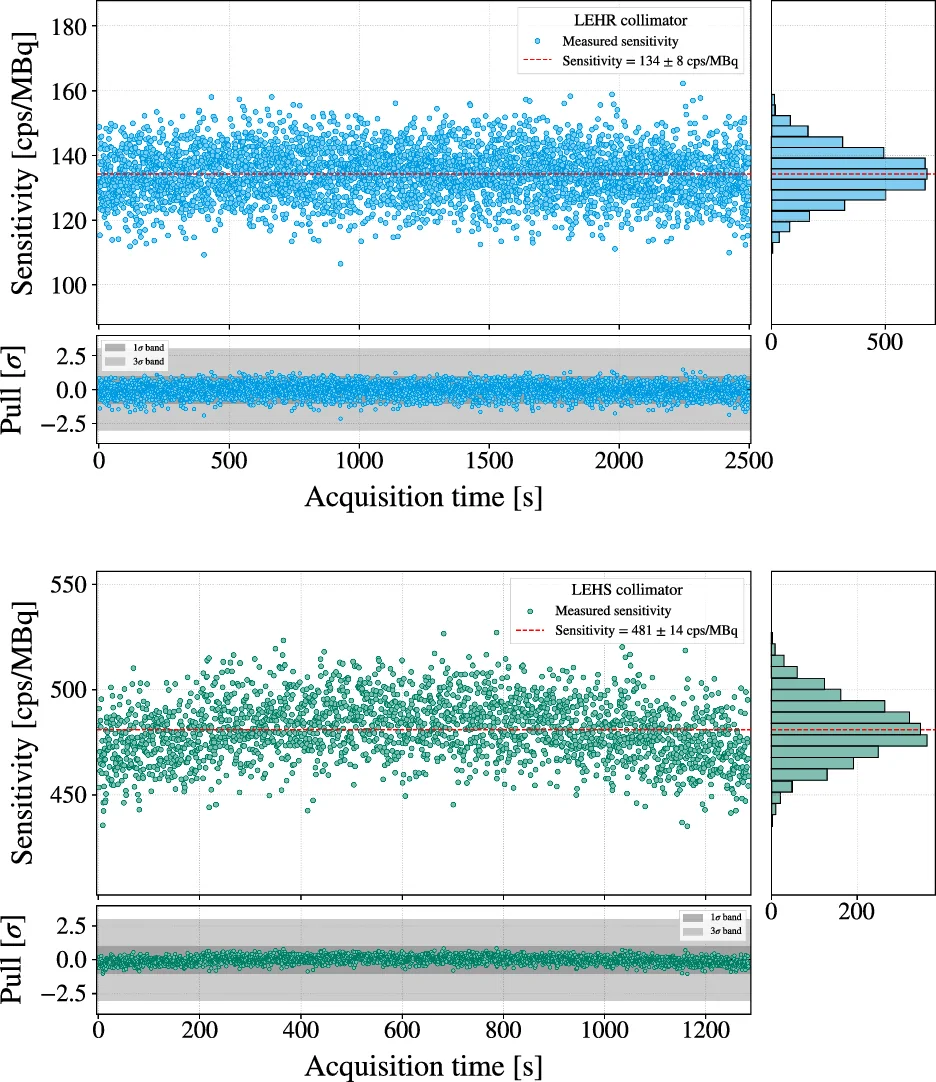
*Top* Sensitivity over time of the camera equipped with the LEHR collimator at 2 cm from the source, after energy window cut (120-160 keV). *Bottom* Sensitivity over time of the camera with the LEHS collimator mounted on at 2 cm from the source, after energy window cut

The results of the sensitivity evaluation are displayed in Fig. 6 for both collimators. With the LEHR collimator, the measured sensitivity of POSiCS was observed to follow a Gaussian distribution around its central value, with a standard deviation of 8 cps/MBq. The mean and the standard deviation were weighted by the points variance. This is displayed in the sensitivity projection on the right side-plot. The Gaussianity of this distribution implies that the negative exponential decay correction to the activity matches the observed rate. This suggests that the fluctuations after the source’s activity compensation are mostly driven by Poissonian shot-noise. Furthermore, from Table 3, we evince that the skewness and Kurtosis of the points distribution (side plot) are compatible with a Gaussian distribution, for which the typical skewness is 0 and the typical kurtosis is 3. For the LEHS collimator, we observe a distribution of sensitivities characterized by a slight positive skewness (indicating a heavier right tail) and a kurtosis slightly below 3, which suggests a more peaked distribution. This is also evident in the time series of measured sensitivities, which shows a positive vertical bending of the estimated sensitivity around 600 s (cf. Fig. 6). Since the kurtosis and skewness analyses still suggest a mostly Gaussian behaviour, we cannot explain this visual bending at this stage as a systematic feature. On the other hand, it is possible that saturation effects due to the high count rate introduce systematic distortions in the time series. This feature will be further investigated. Due to the possibility of saturation, especially at the beginning of the acquisition time (when the source activity is maximal), we consider the measured extrinsic sensitivity of POSICS equipped with the LEHS collimator as a lower limit.

### Energy resolution

**Fig. 7:**
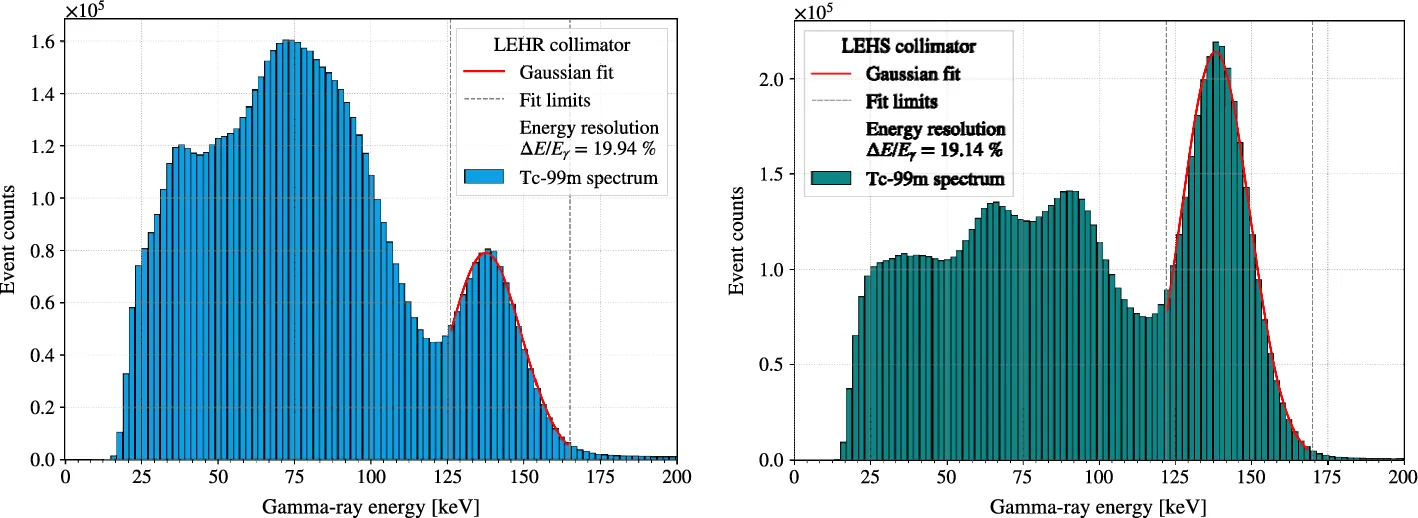
*Left* Corrected spectrum obtained with POSiCS (LEHR collimator) over the whole UFOV. *Right* Corrected spectrum obtained with POSiCS (LEHR collimator) over the whole UFOV. Compton backscattering and Compton edge peaks are visible, before the photopeak at 140 $\textrm{keV}$

The achieved energy resolution with the LEHS collimator is 19.14% ± 0.26%, which aligns with the typical energy resolutions reported in the literature for SFOV gamma cameras [5]. However, this result is relatively sharp for a LYSO-based device [37–39]. This energy resolution is enough to distinguish the photopeak from the nearby structures in the energy spectrum, namely the backscattering peak (located at about 90 keV, as expected) and the Compton edge: the third peak from the Right in Fig. 7.

With the LEHR collimator, it was possible to measure an energy resolution of 19.94% ± 0.20%, which, as expected, is comparable with the resolution achieved through the LEHS collimator. Energy resolution is primarily driven by the scintillator (the number of photons produced per scintillation event), the optical coupling to the photodetector, and the photodetector efficiency. Therefore, as assessed by these tests, the collimator does not significantly impact the energy resolution. However, we notice a large amount of scattered gamma rays with the LEHR collimator, which we link to the increased amount of high-Z scatterer material (tungsten) present due to the longer collimator.

### Collimator lateral leakeage

**Fig. 8:**
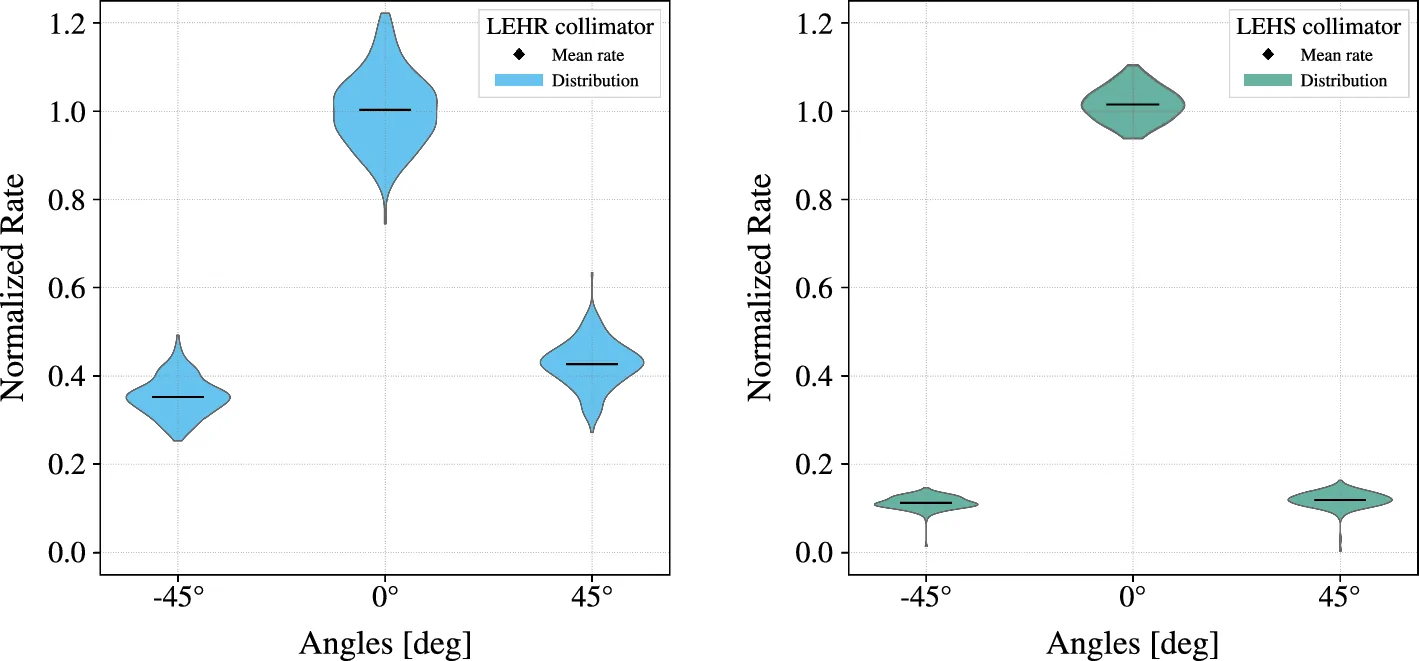
*Left * Leakage test results for the LEHR collimator. *Right * Leakage test results for the LEHS collimator

The tests with both collimators show that the count rate drops significantly when the source is placed outside of the FOV. Moreover, the response is symmetric when placing the source outside of the FOV to the left and Right of the camera. In the case of the LEHS collimator, the average rate drop outside the FOV is 88%, indicating that the shielding around the scintillator effectively eliminates most of the incoming gamma rays. The LEHR collimator shows an average relative rate drop of only 61%. Since both collimators have the same shielding thickness, the observed discrepancy may suggest that the greater amount of tungsten in the LEHR collimator leads to increased gamma-ray scattering. As a result, Compton-scattered gamma rays may still reach the scintillator and be detected. This implies that the energy window cut applied around the photopeak is less effective when using the LEHR collimator. The rate as function of angle is shown in Fig. 8, relative to the the mean rate at $0^\circ$ from the FOV normal direction.

### Camera’s response to other radiotracers ($\mathrm {^{177}Lu}$)

With the device under test, it was possible to observe a $\mathrm {^{177}Lu}$ source emitting at different energies.

The obtained energy spectrum is displayed in Fig. 10. The main emission peak was found to be at 218 keV, which shows, therefore, a 4.6% discrepancy with the theoretical value (208 keV). This behaviour is expected, since the camera was calibrated based on a 140.5 keV reference energy, and assumes linear response of the light yield to the reconstructed charge. For this reason, the energy look-up table (LUT) was rescaled to ensure that the main emission peak was centered at 208 keV.

**Fig. 9:**
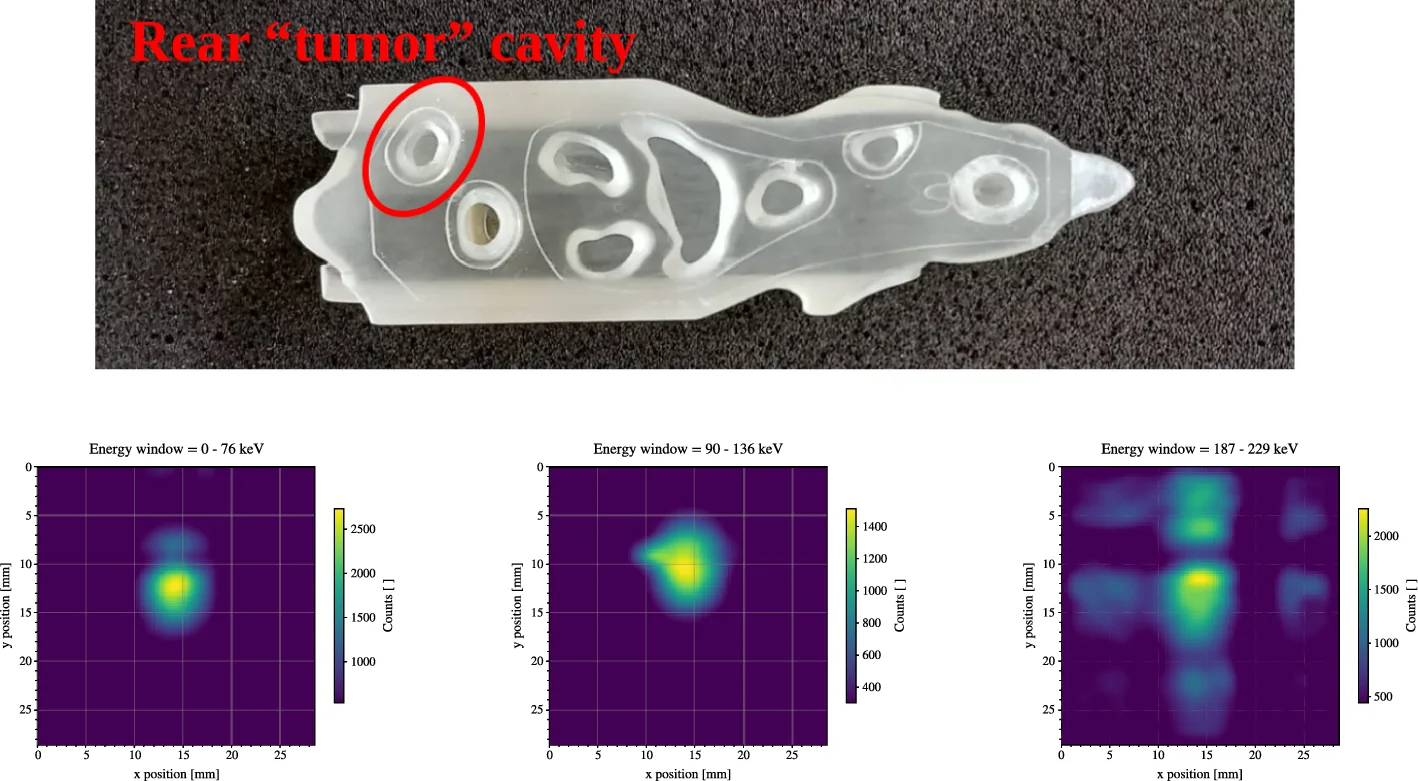
*Top *Bioemtech Mice Phantom used for the $\mathrm {^{177}Lu}$ test. The rear "tumor" cavity was filled with the radiotracer and covered by the phantom’s plastic lid. Phantom from [36]. *Bottom *Reconstructed cavity image for three different energy windows after triple correction (Linearity, Energy cut, and uniformity compensation), treated with Gaussian kernel and spline interpolation

We recognize the first peak as very low-energy X-rays and background radiation, coming from electronics noise and characteristic X-rays from the lead shielding. We identify peak number 2 as the X-ray emissions at 55 keV and 64 keV, which merge into a single peak at 60 keV due to the limited energy resolution of the camera at such low energies, as already observed by [33]. Peak number 3 is the 113 keV emission, which is clearly distinguishable and well centered around its reference value. We identify peak number 4 as the Compton shoulder of the main emission line (peak 5). It is also possible to notice a small bump in the spectrum after the 208 keV energy, which could correspond to a weak emission of $\mathrm {^{177}Lu}$ (321 keV). However, this energy has a low photoelectric absorption probability within the thin scintillator crystal of the POSiCS camera and, therefore, cannot be identified. Moreover, this higher-energy region is also populated by the Lu-176 background due to the presence of this isotope in the LYSO:Ce scintillator (cf. "LYSO scintillator approach" subsection). It should be noted that the spectrum drops to zero below 20 keV due to the energy threshold applied during data acquisition.

**Fig. 10:**
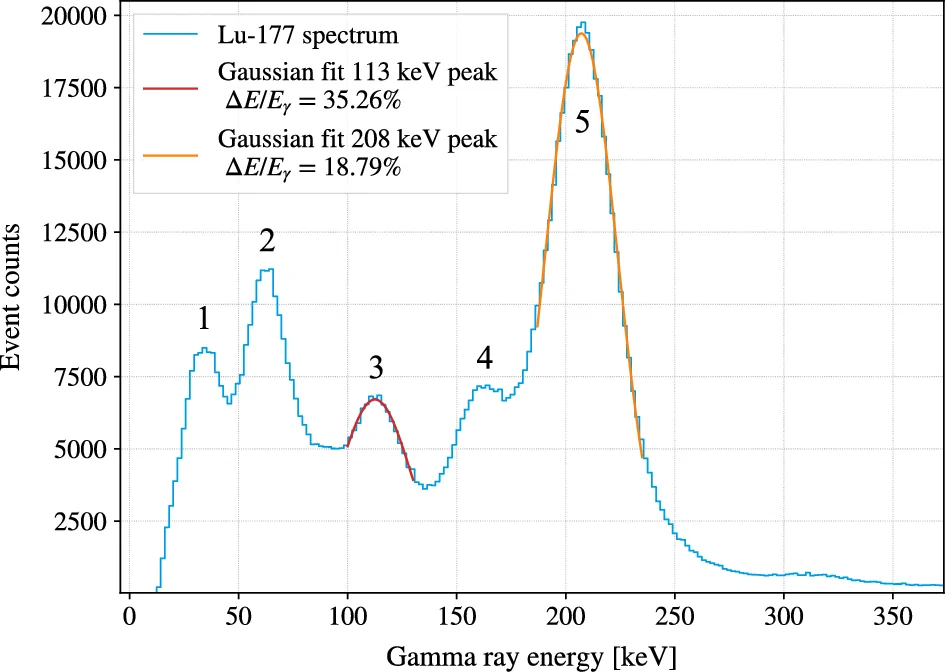
Recorded and rescaled spectrum obtained through the mouse-phantom cavity measurement. The measured energy resolutions at 113 keV and 208 keV are also reported

The primary emission energies used for $\mathrm {^{177}Lu}$ SPECT imaging are the 113 keV and 208 keV gamma-rays. In our case, the energy resolution of these two photopeaks was determined through a Gaussian fit and found to be 35% and 19%, respectively. We therefore proceeded with the usual reconstruction pipeline, by defining two 20% energy windows around these peaks (following the approach of [31]) and a large energy window (0–76 keV) to assess the reconstructed images through low-energy X-ray emission. Those peaks were already taken as a reference for imaging with $\mathrm {^{177}Lu}$ in other works [34, 35].

The imaging results after the standard image correction pipeline, Gaussian kernel convolution (following the NEMA standards guidelines [12]), and spline interpolation, are presented in Fig. 9 (more information about the correction pipeline are reported in "The POSiCS camera" subsection). The cavity image can be efficiently reconstructed for both the X-ray and 113 keV energy windows. However, we encountered significant problems in reliably reconstructing an image at 208 keV. As shown in  Fig. 9, gamma-ray detection in the 208 keV range extends beyond the cavity’s region of interest (ROI), with signals detected in pixels outside the area filled with activity. This issue arises from the use of thin collimator septa, which are optimized for lower energies and fail to collimate gamma rays at 208 keV adequately.

This test demonstrated that the POSiCS camera, without any modification to its configuration or components, is also suitable for imaging with $\mathrm {^{177}Lu}$. It effectively detects events associated with the isotope’s X-ray emissions as well as gamma emissions up to its 113 keV peak. However, imaging at higher energies would require the use of a high energy collimator, either with thicker septa or, equivalently, with longer holes. The performance of the current LEHR collimator with $\mathrm {^{177}Lu}$ is presently under investigation.

## Discussion

### Comparison with existing literature

**Table 4:** Performance parameters results presented in this work

POSiCS camera performance	LEHS collimator	LEHR collimator
*Spatial resolution*		
Spatial resolution AEC (122 keV) @0 cm [mm]$^*$	1.9 ± 0.1	1.4 ± 0.3
Spatial resolution AEC (122 keV) @2 cm [mm]$^*$	4.2 ± 0.1	2.4 ± 0.3
Spatial resolution AEC GC (122 keV) @2 cm [mm]$^*$	5.2 ± 0.1	3.9 ± 0.3
*Sensitivity*		
Sensitivity BEC (140.5 keV) @2 cm [cps/MBq]	1444 ± 31	730 ± 16
Sensitivity AEC (140.5 keV) @2 cm [cps/MBq]$$^{**}$$	481 ± 14	134 ± 8
*Other parameters*		
Energy resolution (140.5 keV) @2 cm	19.14% ± 0.26%	19.94% ± 0.20%
Rate drop outside FOV (122 keV) @3 cm	88%	61%
Weight [kg]	0.313	0.381
Field of view (FOV)	$30.8\times 30.8~\mathrm {mm^2}$	$30.8\times 30.8~\mathrm {mm^2}$
Useful field of view (UFOV)	$28.6\times 28.6~\mathrm {mm^2}$	$28.6\times 28.6~\mathrm {mm^2}$

*Results of resolution measurement with scatterer material. **Lower limit of the sensitivity for this collimator. BEC, before energy cut; AEC, after energy cut; GC, with Gaussian Kernel convolution

In this work, we presented a performance study of the newly developped POSiCS camera. A summary table of these results is presented in Table 4.

To reference POSiCS performances within the landscape of already existing IGCs, a comparison with the literature is proposed, starting with the following parameters: Spatial resolution, extrinsic sensitivity, energy resolution and device’s weight.

**Fig. 11:**
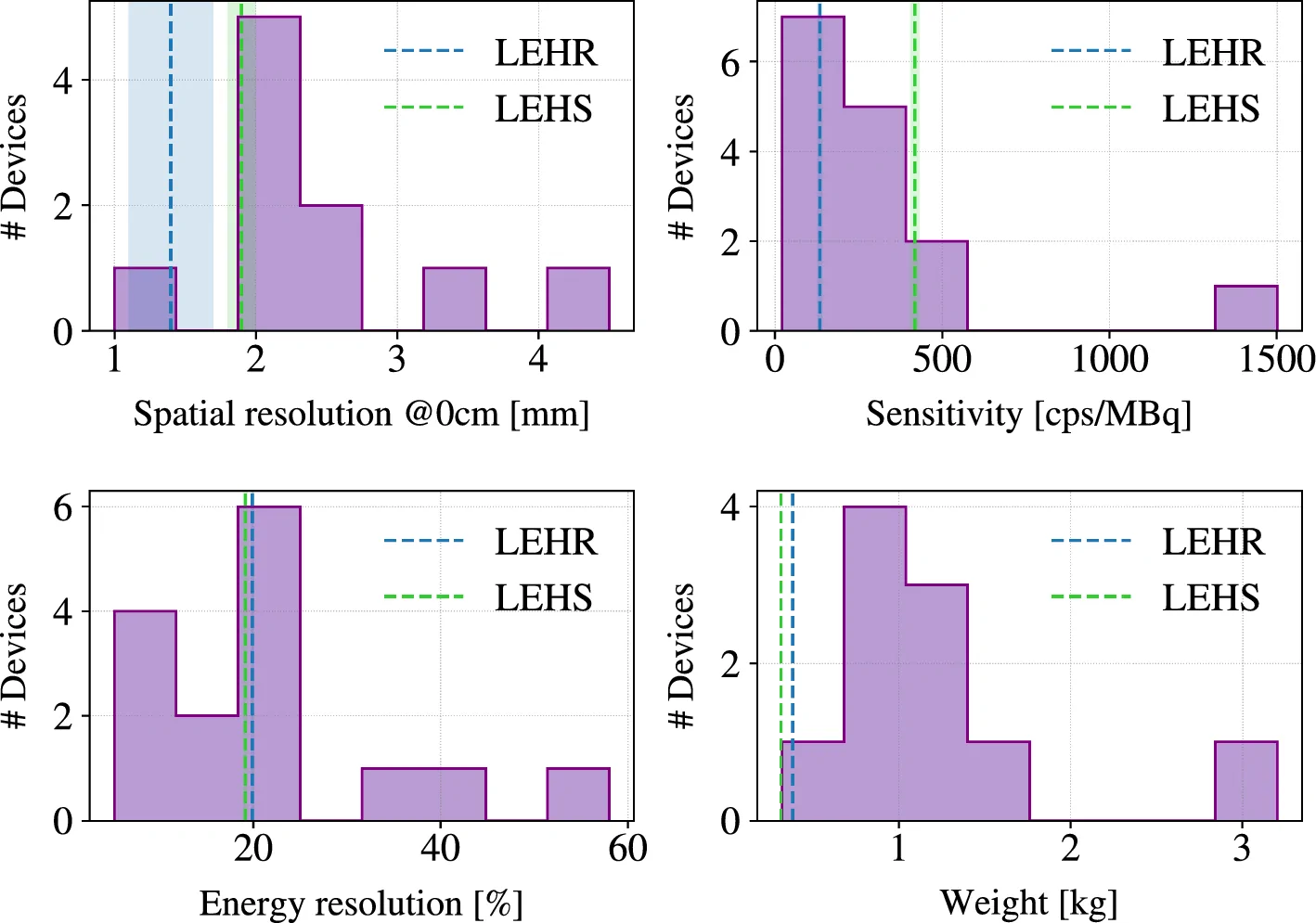
Distribution of the devices performance pareameters repoted in literature, compared to the figure of merits for the POSiCS camera measured in thiw work. The shaded bands represent the experimental uncertainty on POSiCS’ parameters

The evaluation of POSiCS performance parameters can be compared with literature using the comprehensive 2023 review on IGCs [5]. From this review, the relevant performance and physical characteristics of other intraoperative SFOV gamma cameras were extracted. The mean value, the standard deviation, and the minimal and maximal values of these parameters are reported in Table 5. Only devices for which the target parameter was available were included in the statistical analysis and comparison. For cameras featuring multiple operating modes (e.g., interchangeable collimators of different lengths), each parameter was selected from the configuration that most favored the corresponding performance metric, to be conservative. The devices parameters distribution compared to the results of this work for the POSiCS camera are presented in Fig. 11 and commented below.

For spatial resolution, only devices for which the resolution at 0 cm from the source was specified were taken into consideration, in order to be compared with the in-contact performances of POSiCS. It is important to highlight that the evaluation of POSiCS spatial resolution was performed at an energy of 122 keV, while some of the devices used for the comparison evaluated the same parameter at 140 keV. POSiCS shows an extrinsic resolution of a few millimeters even at distances of 4 cm from the target source. This requirement is satisfied with both collimators. The resolution of the camera at 0 cm from the source outperforms 9 devices out of 10 with both the LEHS and LEHR collimators. However, this trend may vary at longer source-to-camera distances due to the geometrical projection effect of the collimator. Overall, POSiCS meets the millimetric spatial resolution requirement up to a source-to-collimator distance of 4 cm, in agreement with the requirements for SLNB.

Since the extrinsic sensitivity of a gamma camera is less dependent on the distance from the source [26], we opted to consider all the devices with known sensitivity estimation, regardles of the reference source-to-collimator distance from the source at which the parameter was estimated.

From Table 5, we notice a large standard deviation of the devices’ sensitivity statistics, which is driven by the different purposes of the cameras presented in the 2023 IGC review (some of which target high resolution, and others high sensitivity). The LEHR camera outperforms only 2 out of 15 devices in terms of sensitivity, since the main target of this configuration is to have a high-precision in image reconstruction, and is therefore more suited for higher activity sources. POSICS with the LEHS collimator, instead, shows a sensitvity well above average, outperforming 12 devices out of 15. Only one device has a significantly higher sensitivtiy (about 1500 cps/MBq). This specific gamma camera leverages a peculiar collimator-scintillator configuration enhancing the device’s sensitivity, and represents a far outlier in the statistics.

The measured energy resolution of the POSiCS camera is in line with the average of 21.4% presented in the review (even if this value, once again, was evaluated at 122 keV and 140 keV, depending on the device). The cameras showing a significantly sharper energy resolution with respect to POSiCS are either scintillator cameras based on scintillators with higher light yields, such as LaBr$_3$:Ce or IGCs performing imaging with a direct detection system, such as CZT detectors that are therefore more suited for spectroscopy. Nevertheless, the observed energy resolution remains competitive, especially when compared to larger LYSO-based systems operating near 140 keV, which often exhibit energy resolutions exceeding 20%. Moreover, LYSO offers additional benefits: its high density allows for the development of compact detectors without reducing the devices’ sensitivity.

A comparison of the cameras’ weights was also performed. This parameter is of great importance to introduce gamma cameras within the protocol of SNLB and ROLL standard procedures, since it allows a easier manipulation of the device. POSiCS outperforms 9 devices out of 10 with the LEHR collimator configuration, while it outperforms all the devices with the LEHS collimator, with a weight well below the average of 1.27 kg. Only one device had a weight comparable to POSiCS (about 320 g), but with a FOV of 1.3*× *1.3 cm$^2$, implying a very small device and hence a reduced weight.

**Table 5:** Summary statistics of the main performance parameters for the analyzed cameras

Parameter	# Devices	Mean	Std. dev.	Min	Max
Spatial resolution @0 cm [mm]	10	2.44	0.95	1.00	4.50
Sensitivity [cps/MBq]	15	316.76	356.20	21.4	1500
Energy resolution [%]	15	21.25	13.74	5.2	58.0
Weight [kg]	10	1.27	0.76	0.32	3.20

### Lateral collimator leakage tests

Shielding tests revealed some residual gamma-ray penetration in both collimator. When outside of the FOV, the camera mounted with the LEHS collimator showed a rate drop of 88%, while in LEHR configuration, the rate drop was of only 61%. We explain this due to the increased amount of scattering associated to the longer collimator (as proven by the full FOV energy spectrum of the camera), and to the lower amount of accepted photons by the LEHR collimator. The violin plots in Fig. 8 show that the distribution of normalized rates is broader for the HR collimator than for the HS configuration. This larger variability is observed because the HR collimator registers a lower absolute rate, as it is designed to reject a larger fraction of incoming trajectories (i.e. reject more gamma-rays). Since the leakage from the camera’s sides is expected to be similar for both collimators (given their identical shielding thickness) the same absolute leakage produces a larger relative contribution in the HR case. As a result, the relative rate reduction is smaller for the HR collimator. This implies that the lateral leakage has a stronger impact on image reconstruction for the LEHR configuration, and might dominate when observing low activity sources within the FOV (for instance when observing near the injection zone).

The analysis shows that an improved collimator design with enhanced lateral shielding could mitigate this penetration effects, and that might be required especially for the HR collimator.

### POSiCS performances at different energies

Tests with $^{177}$Lu were performed due to the increased need for radiation monitoring in $^{177}$Lu-DOTA-TATE and $^{177}$Lu-PSMA procedures, and to test POSiCS performances at different energies.

The tests revealed that it was possible to image a liquid $^{177}$Lu source in a plastic mouse phantom, with the two energy windows: 0–76 keV and 90–136 keV, targeting low energy $\mathrm {^{177}Lu}$ emissions (X-Rays) with the first window and the 113 keV emission line, with the second window. The third emission line, at 208 keV, while composing the larger fraction of detected gamma-rays, was not suited for imaging. The collimator walls of the LEHS collimator were too thin to correctly absorb rejected trajectory, and this results in a strong leakage in the whole camera FOV, as shown in Fig. 9. Tests with the LEHR collimator are currently being performed and the possibility of a $\mathrm {^{177}Lu}$ based collimator with thicker septa is under evaluation.

The higher septal penetration due to the 208 keV emission of $\mathrm {^{177}Lu}$ is a well known problem in SFOV gamma cameras, and has been reported for other devices [34, 35]. Once again, the assessment and evaluation of a $\mathrm {^{177}Lu}$ dedicated collimator could allow the POSiCS camera to provide imaging at 208 keV with the simple need of swapping the device’s head.

## Conclusion

The POSiCS camera was developed as a lightweight, compact, and wireless IGC to assist surgeons during SLNB and ROLL procedures. Integrating rapid, user-friendly imaging systems in surgical settings can reduce procedural invasiveness, thus enhancing both treatment efficacy and postoperative outcomes.

POSiCS demonstrated high spatial resolution when equipped with a LEHR collimator and showed good sensitivity in its LEHS collimator configuration. By comparing the results described in this work with current state-of-the-art IGCs, we demonstrated that POSiCS performances are comparable to, or superior to, currently available systems [4, 5], while offering additional benefits, such as a lightweight module (less than 400 g) and wireless communication. These features significantly improve usability within the surgical environment.

Furthermore, the camera successfully detected gamma emissions from $\mathrm {^{177}Lu}$, demonstrating imaging capability up to the 113 keV photon emission line of this isotope. It was also possible to produce reliable images of the source by detecting lower-energy X-rays. These findings suggest that the device’s field of applications extends beyond traditional techniques involving the administration of $\mathrm {^{99\,m}Tc}$, and may be suited for applications in theranostics and radiotherapeutic dosimetry, without the need for modifications to the detector system.

## Data Availability

The datasets used and/or analysed during the current study are available from the corresponding author on reasonable request.

## Abbreviations

AEC: After energy cut
ADC: Analog-to-digital converter
BEC: Before energy cut
CZT: Cadmium–zinc–telluride
CoG: Center of gravity
DAC: Digital-to-analog converter
FOV: Field of view
FGS: Fluorescence-guided surgery
GATE: Geant4 application for tomographic emission
HR: High-resolution collimator)
HS: High-sensitivity (collimator)
IGC: Intraoperative gamma camera
LEHR: Low-energy high-resolution (collimator)
LEHS: Low-energy high-sensitivity (collimator)
LG-SiPM: Linearly graded silicon photomultiplier
LUT: Look-up table
LYSO:Ce: Lutetium-yttrium oxyorthosilicate, cerium-doped
NEMA: National electrical manufacturers association
PET: Positron emission tomography
PMMA: Polymethyl methacrylate
POSiCS: Position-sensitive SiPM compact and scalable gamma-camera
PS: Position-sensitive
RGS: Radio-guided surgery
ROI: Region of interest
ROLL: Radioguided occult lesion localization
SC: Slow control
SPECT: Single-photon emission computed tomography
SiPM: Silicon photomultiplier
SLNB: Sentinel lymph node biopsy
SPAD: Single-photon avalanche diode
TIA: Transimpedance amplifier
UFOV: Useful field of view
